# Adverse childhood experiences and fronto-subcortical structures in the developing brain

**DOI:** 10.3389/fpsyt.2022.955871

**Published:** 2022-10-06

**Authors:** Elizabeth E. L. Buimer, Rachel M. Brouwer, René C. W. Mandl, Pascal Pas, Hugo G. Schnack, Hilleke E. Hulshoff Pol

**Affiliations:** ^1^UMC Utrecht Brain Center, University Medical Center Utrecht, Utrecht University, Utrecht, Netherlands; ^2^Department of Complex Trait Genetics, Centre for Neurogenomics and Cognitive Research, VU University Amsterdam, Amsterdam, Netherlands; ^3^Experimental Psychology, Utrecht University, Utrecht, Netherlands; ^4^Department of Languages, Literature and Communication, Faculty of Humanities, Utrecht University, Utrecht, Netherlands; ^5^Department of Experimental Psychology, Helmholtz Institute, Utrecht University, Utrecht, Netherlands

**Keywords:** adverse childhood experiences, child, brain structure, stress, neuroimaging, YOUth cohort study

## Abstract

The impact of adverse childhood experiences (ACEs) differs between individuals and depends on the type and timing of the ACE. The aim of this study was to assess the relation between various recently occurred ACEs and morphology in the developing brain of children between 8 and 11 years of age. We measured subcortical volumes, cortical thickness, cortical surface area and fractional anisotropy in regions of interest in brain scans acquired in 1,184 children from the YOUth cohort. ACEs were based on parent-reports of recent experiences and included: financial problems; parental mental health problems; physical health problems in the family; substance abuse in the family; trouble with police, justice or child protective services; change in household composition; change in housing; bereavement; divorce or conflict in the family; exposure to violence in the family and bullying victimization. We ran separate linear models for each ACE and each brain measure. Results were adjusted for the false discovery rate across regions of interest. ACEs were reported for 83% of children in the past year. Children were on average exposed to two ACEs. Substance abuse in the household was associated with larger cortical surface area in the left superior frontal gyrus, *t*(781) = 3.724, *p*_*FDR*_ = 0.0077, right superior frontal gyrus, *t*(781) = 3.409, *p*_*FDR*_ = 0.0110, left pars triangularis, *t*(781) = 3.614, *p*_*FDR*_ = 0.0077, left rostral middle frontal gyrus, *t*(781) = 3.163, *p*_*FDR*_ = 0.0195 and right caudal anterior cingulate gyrus, *t*(781) = 2.918, *p*_*FDR*_ = 0.0348. Household exposure to violence (was associated with lower fractional anisotropy in the left and right cingulum bundle hippocampus region *t*(697) = −3.154, *p*_*FDR*_ = 0.0101 and *t*(697) = −3.401, *p*_*FDR*_ = 0.0085, respectively. Lower household incomes were more prevalent when parents reported exposure to violence and the mean parental education in years was lower when parents reported substance abuse in the family. No other significant associations with brain structures were found. Longer intervals between adversity and brain measurements and longitudinal measurements may reveal whether more evidence for the impact of ACEs on brain development will emerge later in life.

## Introduction

A history of adverse childhood experiences (ACEs), such as maltreatment, parental divorce, exposure to violence or substance abuse in the family, is a risk factor for developing mental health problems later in life (1–3). ACEs are associated with decreased life expectancy, for example *via* the effects of ACEs on toxic stress, increased adult health risk behavior, increased suicidality or socioeconomic inequality (4–8). These studies also show that especially individuals exposed to cumulative adversity are at risk for mental or physical health problems later in life.

Individual differences explain how a child is impacted by ACEs. Many individuals exposed to adversity are resilient to negative effects. For example, high psychosocial functioning despite a history of childhood maltreatment can be explained by neurobiological and genetic factors, but the social environment can serve as a protective factor as well (9). Even more, volume alterations found in older adults exposed to ACEs vary based on their serotonergic genetic vulnerability (10).

The impact of ACEs also depends on the timing of the ACE in relation to sensitive periods of brain development (11–15). During childhood and adolescence, the brain undergoes considerable developmental changes, including a thinning of the cortex, an increase followed by a decrease in cortical surface area and continued growth of volume of the white matter connections (16–18); and these changes have been related to cognitive functioning (19). Many psychiatric disorders emerge during adolescence (20). Studying how ACEs interact with brain development is crucial to better understand mechanisms of latent vulnerability (21) and resilience (22).

Neuroimaging studies on childhood adversity started with a strong focus on the effects of severe early caregiver adversity, for example in institutionalized children or children exposed to childhood maltreatment. Furthermore, most studies focused on the fronto-limbic network (frontal cortex, hippocampus and amygdala) because of the well-established role of fronto-limbic regions in the hypothalamic-pituitary-adrenal (HPA) axis functioning in response to stress (23), although a meta-analysis concluded that there is no evidence for abnormalities in the amygdala and only weak evidence for smaller hippocampal volumes in adults that experienced childhood adversity (24). There are only a few whole-brain studies on the association between childhood maltreatment and brain structure. Taking meta-analyses and reviews together, the most consistent findings are in fronto-limbic regions, fronto-striatal regions, fronto-subcortical association fibers and the corpus callosum (24–30). Still, the spatial overlap between studies is weak, because most studies rely on smaller samples or adult samples, as children with experiences of maltreatment are difficult to include in large numbers. Furthermore, there is evidence for differential structural brain correlates depending on the types of ACE (31). Therefore, studying a variety of ACEs in developmental populations may shed light on the effects of ACEs on brain development.

In the current study, we investigate the effect of ACEs on brain structure in pre-adolescent children, participating in the first wave of the YOUth cohort study, a longitudinal study where each measurement wave covers a narrow period of development. The main question of the current study is: Is there an association between ACEs and brain structure in pre-adolescent children? Regions of interest were selected *a priori* by integrating studies on structural, functional and neurocognitive correlates of childhood adversity. We focused on subcortical volume, cortical surface area, cortical thickness and fractional anisotropy (FA). The latter was selected as white matter measure because it represents a good measure for integrity of the white matter and has shown good test-retest reliability using our acquisition protocol (32). We expect that our sample size allows for detection of more subtle effects even though the sample is not enriched for children with severe adverse experiences. Within the group of children that experienced adversity, we expect more pronounced effects in children that were exposed to accumulated ACEs, compared to children exposed to a single ACE. Based on the stress acceleration hypothesis (33), we hypothesized that brain development in children exposed to childhood adversity would be ahead of peers. Based on brain development curves in previous studies, we expect that in 8-, 9-, and 10-year-olds accelerated development would mean thinner cortices (34), larger subcortical volumes (35), large cortical surface area (36), and larger FA (37). Age- and sex-effects on global brain measures are included as well, to provide a full description of the YOUth cohort sample for comparison with other cohorts.

## Materials and methods

### Participants

We included 1,184 children that participated in the first wave of the population-based longitudinal YOUth cohort study. The cohort rationale, design and procedures are described in detail elsewhere (38). In short, participants are living in the province of Utrecht (Netherlands) and its surrounding areas, a densely populated region that combines both urban and rural areas. Compared to the rest of the Netherlands, inhabitants of the province of Utrecht are relatively highly educated. Most children were recruited through their primary school. YOUth includes children and their parents. Parents are considered those with parental authority over the child. Children are excluded if they are not mentally or physically not capable of participating, if they or their parents’ language proficiency in Dutch is not sufficient to understand provided information. For the neuroimaging part of the study, we excluded children with metal implants including most braces, following fairly standard MRI procedures. Participating children were between 7.9 and 11.0 years old (56% females). All data was collected prior to the COVID-19 pandemic. The study was approved by the Medical Research Ethics Committee Utrecht. Children participated on a voluntary basis and parents or guardians gave written consent and assent.

### Data on adverse childhood experiences

Adverse childhood experiences were collected using parent reports on life events that occurred in the household in the past year, available for 1,046 children. From the recent life events questionnaire, we selected nine types of ACEs: financial problems; physical health problems in the family; substance abuse in the family; trouble with police, justice or child protective services (CPS); change in household composition; change in housing; bereavement; divorce or conflict in the family; exposure to violence in the family. In addition, two ACEs were gathered from other questionnaires as they were not covered in the recent life events survey. First, information on bullying behavior toward the child was available for 948 children. From the bullying questionnaire one ACE was created by selecting whether children were exposed to any type of frequent bullying at least one time a week. Second, information on parental psychiatric diagnoses was available for 1,056 children. Parental mental health problems were indicated as an ACE if one or more parents or guardians were diagnosed with at least one psychiatric diagnosis.

All ACEs were used as binary variables (yes/no). In most cases, information from different questionnaire items was combined into a single composite variable. For example, change in household composition would be set to yes if at least one new member was added to the household, for example cohabitation of a new partner, cohabitation of a step brother or sister, birth of a new family member etcetera. As these separate items are conceptually very similar, we regard this as a single event rather than multiple independent events. Overlap between the separate items of the recent life events survey was mapped (Supplementary Figure 1), but not decisive when creating the composite variables, because high overlap between items does not necessarily implicate a single underlying environmental factor. Overlap was mapped by taking the subgroup of children that experienced a specific event and then computing the percentage of the children in this subgroup that additionally experienced another event. In the same way, we mapped the overlap between the final 11 ACEs used in this study (Figure 2C).

The prevalence of ACEs was similar in the total group, compared to the subgroups that had MR data available. Because we used various data sources for the ACEs, sample sizes differed between bullying, recent life events and parental psychiatric diagnosis dependent on the overlap of respondents with available MR data.

### Image acquisition

The collection of MRI data is closely monitored in the YOUth cohort study. Patterns in data quality are monitored over time based on human data and weekly collected phantom data. The YOUth MRI protocol, quality control and test-retest reliability are described in detail elsewhere (32). In short, anatomical T1-weighted MRI scans were available for 956 children and diffusion-weighted images (DWI) for 895 children. All MR scans were acquired on the same scanner, a Philips Ingenia 3.0 T CX scanner with a 60 cm bore (Philips Medical Systems, Best, Netherlands) using a 32- channel SENSE head-coil. A structural T1-weighted 3D gradient echo scan was acquired with the following parameters: TR = 10 ms; TE = 4.6 ms; flip angle = 8°; reconstructed voxel size = 0.75 mm× 0.75 mm× 0.80 mm; parallel imaging factor = 1.70 (AP) and 1.40 (RL). Next, a diffusion-weighted multi-shell multi-band echo planar (EPI) acquisition is obtained including two short DWI scans with a reversed phase encoding readout to correct for susceptibility artifacts. The following parameters were used to acquire the DWI scan: TR = 3500 ms; TE = 99 ms; flip angle = 90°; reconstructed voxel size = 2.0 mm× 2.0 mm× 2.0 mm; multiband acceleration factor = 3; parallel imaging factor = 1.3; b-values = 500 (15), 1000 (30), 2000 (39) and every 10th scan is a diffusion unweighted (b-value = 0) scan. At the start of the study a different DWI protocol was used. Therefore, 13% of the included data was acquired without reversed phase encoding readout and with the following parameters: TR = 6827 ms; TE = 101 ms; flip angle = 90°; reconstructed voxel size = 2.5 mm× 2.5 mm× 2.5 mm; parallel imaging factor = 2.5; b-values = 1000 (15), 2000 (25), 3000 (35) and every 10th scan is a diffusion unweighted scan. We corrected for the different protocols in our analyses (see below for details).

### Image processing

At the time YOUth provided access to the data for this study, defacing or face masking procedures had not yet been implemented. Therefore, T1-weighted scans were not subjected to any defacing or face masking procedures that may have a small effect on outcome measures (40).

FreeSurfer 6.0 was used for automatic brain segmentation and parcellation of the T1-weighted scans (41). Subcortical data was extracted from the output of FreeSurfer’s volume-based stream. For the initial registration and non-linear alignment steps we relied on the default MNI305 atlas. Cortical data was extracted from the output of FreeSurfer’s cortical surface-based atlas. For registration of the individual surfaces to an average sphere we used the default fsaverage. The Desikan-Killiany atlas was used for cortical parcellation (42). To be able to study whether our findings were specific to the ROIs rather than a global effect, we also extracted intracranial volume and computed total cortical surface and average cortical thickness.

Diffusion-weighted images scans were processed using FSL version 6.0.1 (43) in combination with MRtrix 3.0 (44). The processing pipeline consisted of denoising (45, 46), gradient direction corrections (47), eddy current corrections (48), susceptibility corrections (49) and corrections for Gibbs-ringing artifacts (50). FSL’s EDDY QC framework was used to get quality control reports for each individual (QUAD) and at the group-level (SQUAD) (48, 51–53). QC parameters in the QUAD output include estimates of absolute motion, relative motion, translations, rotations, eddy current linear terms, susceptibility, B-value outliers, signal-to-noise ratio and contrast-to-noise ratio. Next, FSL’s Tract-Based Spatial Statistics (TBSS) was used to skeletonize the fractional anisotropy (FA) maps in standard space (53, 54). For the TBSS registration and transformation to MNI152 space we used the default adult template, FMRIB58_FA. Using the TBSS processing pipeline we also generated a webpage with, for each individual, slices of the FA maps for visual quality control. Lastly, the intersection between the skeleton and the regions of the JHU-ICBM-DTI-81 atlas (55) was used to compute the average FA values for these regions. Furthermore, the mean FA over all atlas regions was computed.

### Quality control

For the T1-weighted scans, an experienced rater visually assessed image quality for each individual based on the original scan and segmentation quality based on the pial reconstruction. From the 956 T1-weighted scans a total of 132 scans were excluded for various reasons: motion artifacts that affected pial surface reconstruction (*N* = 96), inhomogeneity artifacts (*N* = 17), brain anomalies (*N* = 9), FreeSurfer failed (*N* = 5), corrupt DICOM files (*N* = 3), dental artifact (*N* = 1), and incorrect field-of-view (*N* = 1). This resulted in gray matter estimates for 824 children.

For the DWI scans, we started off with 895 scans. We used the presence of artifacts on the T1-weighted scans as a predictor for the quality of the DWI scans, thereby excluding the same children that were excluded in the T1-weighted analysis (*N* = 123). Furthermore, DWI data was incomplete or missing in some cases (*N* = 10) or failed the processing pipeline (*N* = 17). Next, based on the visual inspection of the FA map snapshots for each individual, we additionally excluded DWI scans that were acquired in a different orientation (*N* = 7) or with an incorrect field-of-view (*N* = 2). The distribution of the SQUAD QC parameters was as expected. Outliers were visually checked once again, but did not lead to exclusions. We performed t-tests for each QC parameter using the individual-based QUAD output, to test if image quality differed between children without any ACE versus children with at least one ACE. The prevalence of differences between the QC parameters in children with versus without an ACE were as would be expected by chance, with no consistent patterns of lower quality for specific ACE subsets. Therefore, no exclusions were made based on these parameters. The results of FSL’s TBSS were visually checked as recommended in the user guide.^1^ This QC did not result in additional exclusions.

### Regions of interest

**FIGURE 1:**
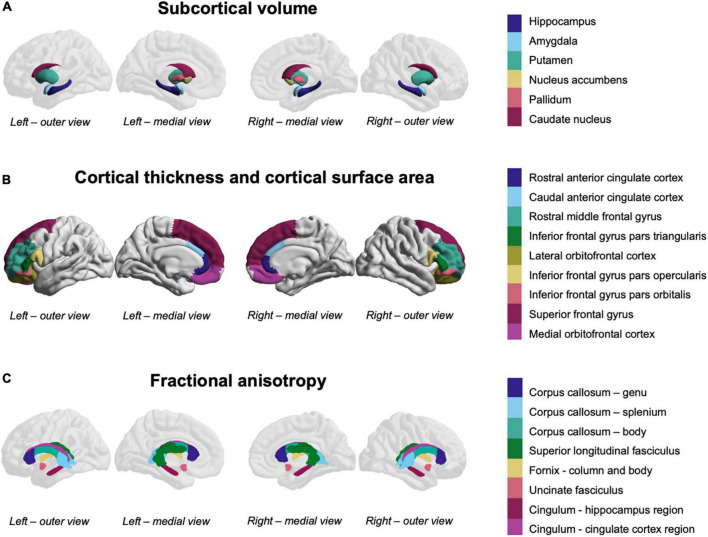
Fronto-limbic and fronto-striatal regions-of-interest. **(A)** Subcortical volume was measured in the colored subcortical structures (overlaid on a reference brain for orientation). **(B)** Cortical thickness and cortical surface area were measured in the colored cortical structures. **(C)** Fractional anisotropy was measured in the colored white matter structures (overlaid on a reference brain for orientation).

Regions of interest were selected from the gray matter and white matter atlases (Figure 1) based on meta-analyses and reviews described earlier (24–30).

**FIGURE 2:**
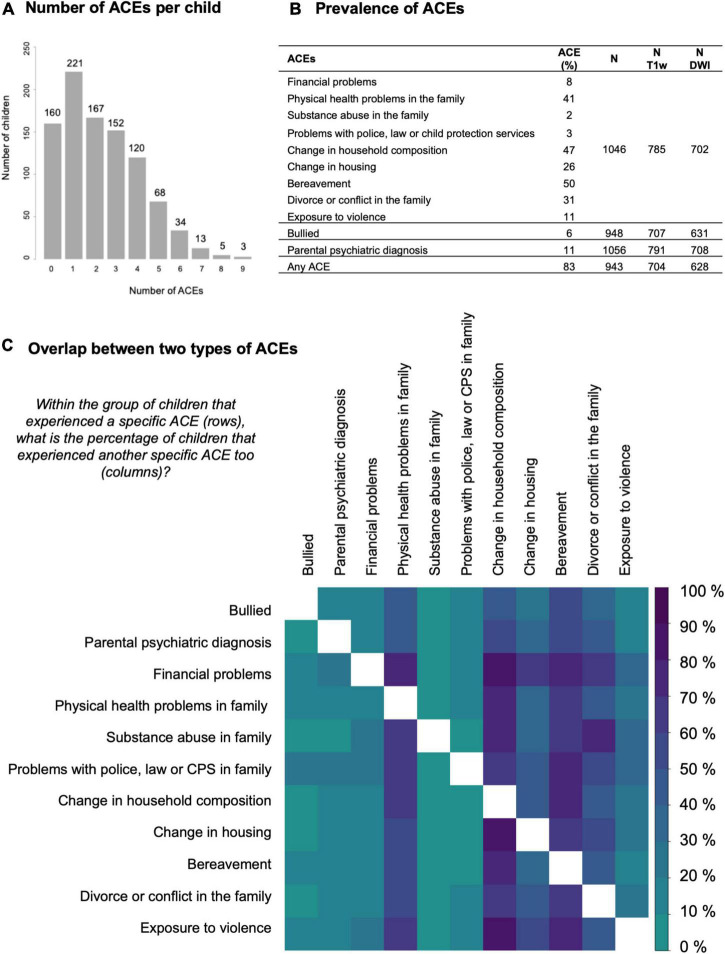
Descriptive statistics for the exposure to ACEs. **(A)** Histogram of the number of adverse childhood experiences (ACEs) per child. **(B)** Table with the prevalence of specific ACEs in our sample. Borders between the rows indicate a different data source: 9 ACEs from the life events survey, 1 from the bullying survey, 1 from the health survey, and 1 composed from all three surveys. The prevalence of ACEs was similar in the total group compared to the subsets with MR data available. The last columns list the sample size for each data source in the total group and in the subsets with T1-weighted MRI data or diffusion-weighted imaging data available. **(C)** Matrix visualizing the overlap between two types of ACEs. Darker colors represent a higher overlap (in percentage) between children with one ACE (row) and a second ACE (column). The upper and lower triangle are not symmetric because the percentage is based on the subset defined in the row. The diagonal is masked out (white).

### Statistical analyses

Using R version 4.0.5 (2021-03-31) in R studio version 1.4.1106 each brain measure was regressed on each ACE in a separate linear model. All variables were scaled and centered to create standardized output that is comparable across cohorts and across different brain measures. Apart from each specific ACE, we also tested the relation between brain measures and any ACE and accumulated ACEs (sum of ACEs per child). Any ACE and accumulated ACE variables were only computed if data was available for all ACEs. All analyses were corrected for age and sex. For the DWI analyses we also included a dichotomous variable to correct for the DWI acquisition protocol. Additionally, to assess regional specificity, we repeated the main analysis for the T1-weighted brain measures correcting subcortical volumes for intracranial volume, regional cortical thickness for average cortical thickness and regional cortical surface area for total cortical surface area.

We corrected for multiple comparisons by controlling the false discovery rate (FDR) (56). Throughout this manuscript we will report uncorrected *p*-values (*p*_*uncorr*_) and FDR-adjusted p-values (*p*_*FDR*_). FDR-adjusted *p*-values were adjusted across T1-weighted or DWI brain measures independently for each ACE separately and thus not across all analyses.

To estimate the robustness of the regression coefficients, we applied non-parametric bootstrapping, drawing random samples with replacement from the residuals of the regression model and added these to the original fitted values to create 5,000 new samples. We chose resampling residuals over resampling subjects because of the small groups for some ACEs.

### Data visualization

For (sub)cortical surface data visualization, we used the ENIGMA toolbox (57). For white matter tract visualization, we used Surfice.^2^ Subcortical and white matter structures were overlaid on a reference brain to indicate the orientation of the structures and the approximate location in the brain.

## Results

### Exposure to adverse childhood experiences

On average children were exposed to two ACEs in the last year and up to nine accumulated ACEs (Figure 2A). The percentage of children with at least one ACE was 83% (Figure 2B). In general, the overlap between different ACEs was as expected based on the prevalence in the group as whole, i.e., for most ACE subgroups the co-occurrence of physical health problems, change in household composition, change in housing, bereavement and divorce or conflict in the family was high. Children growing up in families with financial problems appear to be disproportionately burdened by accumulated ACEs (Figure 2C).

### Age and sex effects for global brain structure

**FIGURE 3:**
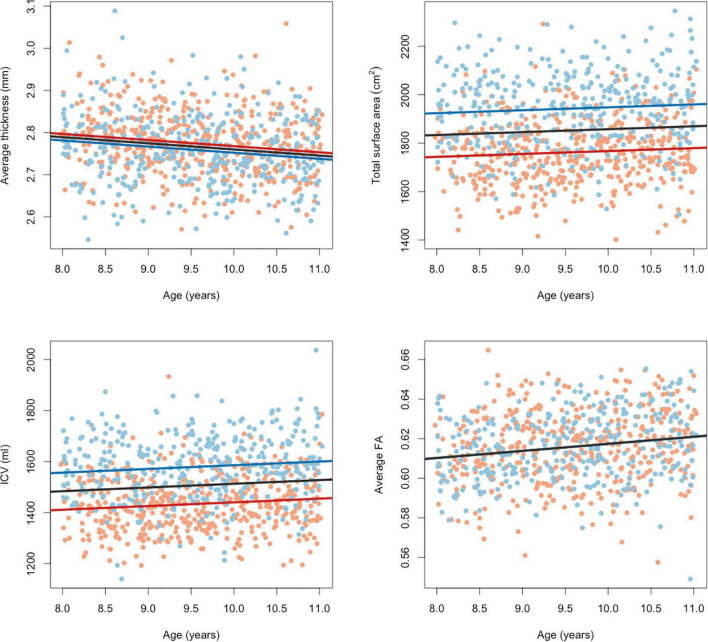
Effects of age and sex on global brain measures. Red dots indicate brain measures in girls and blue dots indicate brain measures in boys. Lines show the relation modeled linearly between brain measures and age (for girls in red, boys in blue and in black for the group as whole). Fractional anisotropy measures were corrected for the different acquisition protocols.

For all brain estimates individual differences were large with overlap between children of different ages and sexes. Still, age effects were found for all global brain estimates and sex effects for all global estimates except average FA (Table 1 and Figure 3). ICV was also positively associated with age *t*(821) = 3.096, *p*_*uncorr*_ = 0.0020, β = 14.556, CI_95%_ [5.328, 23.784] and larger for boys *t*(821) = −17.964, *p_*uncorr*_* < 0.0001, β = −145.426, CI_95%_ [−161.317, −129.536]. Total cortical surface area was also positively associated with age *t*(821) = −2.076, *p*_*uncorr*_ = 0.0382, β = 12.025, CI_95%_ [0.656, 23.395] and larger for boys *t*(821) = −18.109, *p*_*uncorr*_ < 0.0001, β = −180.631, CI_95%_ [−200.209, −161.052]. Total average thickness was negatively associated with age *t*(821) = −4.580, *p*_*uncorr*_ < 0.0001, β = −0.015, CI_95%_ [−0.021, −0.0008] and was on average lower for boys compared to girls *t*(821) = 2.830, *p*_*uncorr*_ = 0.0048, β = 0.015, CI_95%_ [0.005, 0.026]. Average FA over the TBSS skeleton was positively associated with age *t*(732) = 4.732, *p*_*uncorr*_ < 0.0001, β = 0.0036, CI_95%_ [0.002, 0.005] and not significantly associated with sex *t*(732) = 0.101, *p*_*uncorr*_ = 0.9194, β = 0.004, CI_95%_ [−0.002, 0.002]. Age effects were modeled in a linear fashion only. Age squared did not reach significance in the sample’s narrow age range.

**TABLE 1:** Sample characteristics.

	All available data
*Sex (% girls)*	*N* = *955*
	56
*Mean age in years (SD)*	*N* = *955*
	9.54 (0.86)
*Mean CBCL total problem score (SD)*	*N* = *1055*
	22.92 (16.49)
*Self-reported ethnicity mother (%)*	*N* = *1212*
Dutch	91
Dutch and another ethnicity	2
Another ethnicity	7
*Self-reported ethnicity father (%)*	*N* = *955*
Dutch	93
Dutch and another ethnicity	2
Another ethnicity	5
*Mean education in years mother (SD)*	*N* = *1212*
	15.12 (2.00)
*Mean education in years father (SD)*	*N* = *955*
	14.86 (2.50)
*Gross monthly household income (%)*	*N* = *1133*
< €1.250	2
€1.250–€2.000	6
€2.000–€3.000	8
€3.000–€4.000	18
> €4.000	66
*Number of children at home (%)*	*N* = *1232*
0 or 1	12
2	53
3 or more	35

### The association between adverse childhood experiences and brain structure

When focusing only on the standardized effect sizes (β) and ignoring statistical significance, we observe a pattern of larger subcortical volume and larger cortical surface area in children exposed to ACEs. For fractional anisotropy and cortical thickness, results were more mixed. The results for all analyses, sorted by FDR-adjusted p-values and with color-coded effect sizes, can be found in Supplementary Tables 1–5. For most ACEs, none of the ROIs reached significance (*p*_*FDR*_ < 0.05), except for children growing up in a family where substance abuse is an issue and children that grow up in an environment where the parents report exposure to violence.

**FIGURE 4:**
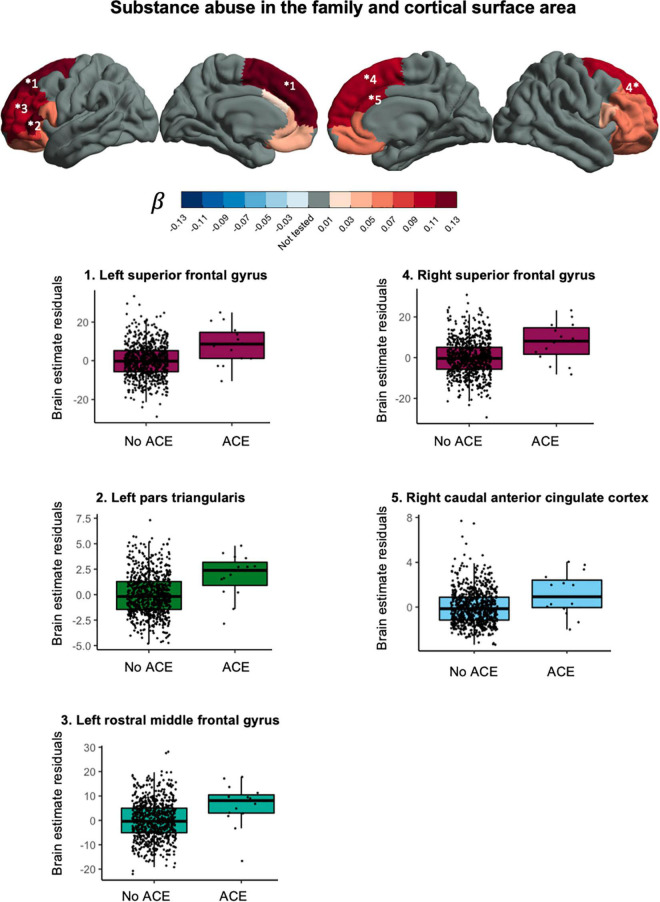
Associations between substance abuse in the household and cortical surface area.

Substance abuse in the household was associated with larger cortical surface area in the left superior frontal gyrus, *t*(781) = 3.724, *p*_*FDR*_ = 0.0077, *p*_*uncorr*_ = 0.0002, β = −0.118, CI_95%_ [0.056, −0.181], right superior frontal gyrus, *t*(781) = 3.409, *p*_*FDR*_ = 0.0110, *p*_*uncorr*_ = 0.0007, β = −0.109, CI_95%_ [0.046, 0.172], left pars triangularis, *t*(781) = 3.614, *p*_*FDR*_ = 0.0077, *p*_*uncorr*_ = 0.0003, β = −0.121, CI_95%_ [0.055, 0.187], left rostral middle frontal gyrus, *t*(781) = 3.163, *p*_*FDR*_ = 0.0195, *p*_*uncorr*_ = 0.0016, β = −0.101, CI_95%_ [0.038, 0.164], and right caudal anterior cingulate gyrus, *t*(781) = 2.918, *p*_*FDR*_ = 0.0348, *p*_*uncorr*_ = 0.0036, β = −0.100, CI_95%_ [0.033, 0.168]. After correction for total cortical surface area, effects were attenuated and no longer significant. Effects in the same direction were found in non-significant ROI’s. Together, this suggest a more global effect on (frontal) cortical surface area. Figure 4 shows the effect sizes and scatter plots for the association between substance abuse in the household and cortical surface area.

**FIGURE 5:**
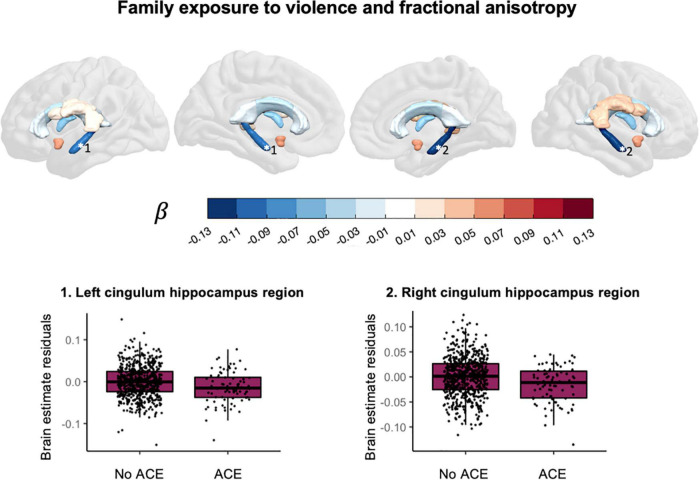
Associations between exposure to violence and fractional anisotropy.

Household exposure to violence was associated with lower fractional anisotropy in the left cingulum bundle hippocampus region, *t*(697) = −3.154, *p*_*FDR*_ = 0.0101, *p*_*uncorr*_ = 0.0017, β = −0.102, CI_95%_ [−0.166, −0.039], and in the right cingulum bundle hippocampus region, *t*(697) = −3.401, *p*_*FDR*_ = 0.0085, *p*_*uncorr*_ = 0.0007, β = −0.121, CI_95%_ [−0.191, −0.051]. The direction of effect in other ROIs was mixed. Figure 5 shows the effect sizes and scatter plots for the association between exposure to violence and FA.

The main results were robust as suggested by comparable means and confidence intervals for the bootstrapping of each regression coefficient. Histograms followed a normal distribution (Supplementary Figures 2–8 and Supplementary Tables 6–12). Bootstrapping confidence intervals for all regression coefficients are reported in Supplementary material.

### *Post-hoc* description of sample subsets

In deviation of our data request (specified in advance of the study) and after the data were seen, we wanted to investigate whether our results could be related to attrition bias and whether our main effects could be explained by other environmental factors. The Supplementary material provides the methods related to this section. Household income was analyzed in the same way as in a previous study on socioeconomic status in, among others, the YOUth cohort (58). Sample characteristics can be found in Table 1. First, children with data on T1-weighted brain measures and recent life events were compared to children with missing data for either the T1-weighted brain measures or the life events survey. Children with missing data scored on average two points higher on the CBCL total problems score (Supplementary Table 13). The same was found for children with missing DWI data or life events data and, additionally, the percentage of fathers with another self-reported ethnicity than Dutch was higher in this group with missing data (Supplementary Table 14). Second, subsamples with and without ACEs were compared for the ACEs and brain scans relevant for our main effects. We found that parents’ educational attainment in years was shorter for children with versus without exposure to substance abuse in the family (Supplementary Table 15) and lower household incomes were more prevalent when parents reported exposure to violence (Supplementary Table 16).

## Discussion

This study explored the association between various ACEs and brain morphometry in selected ROIs during pre-adolescence in a cohort of over 1,000 children between 7 and 11 years old. We found an association between substance abuse in the household and larger cortical surface area in frontal regions (Figure 4). Furthermore, we found evidence for an association between exposure to violence and lower fractional anisotropy in the bilateral cingulum bundle in the hippocampus region (Figure 5).

This study contributes to previous work by providing specific ACEs that could be worth further investigation: substance abuse in the family and exposure to violence. Growing up in a family with substance abuse problems was associated with a larger cortical surface area in the bilateral superior frontal gyrus, the left pars triangularis, the left rostral middle frontal gyrus and the right caudal anterior cingulate gyrus. A previous study in pre-adolescent children found an association between growing up in a family with substance abuse problems and larger cortical surface area in frontal regions (59). In that same study, thinner cortices were also found and we did not replicate that finding. For white matter we found an association between family exposure to violence and lower fractional anisotropy in the bilateral cingulum bundle hippocampus region. The cingulum is the tract that connects the frontal cortex with the parahippocampal gyrus in the temporal lobe. Exposure to violence has in the past been associated with lower quantitative anisotropy in the hippocampal cingulum (60) and lower mean diffusivity but not difference in fractional anisotropy in the hippocampal cingulum (39).

In general, we found small effects and no evidence for significant differences in most ACEs. There are several explanations to be considered. One, there is discussion as to what qualifies as an ACE. We rely on parent-report and do not know to what extent children were impacted by the ACEs. Two, only ACEs within one year before the first wave were measured. Thus, effects of ACEs that occurred before that time and may have influenced brain development could not be included in the analysis. Effects of recent ACEs on brain morphology may emerge later in development. Three, the effects of ACEs on the brain could be too small to detect with our current method. Rather than running separate analyses for each brain measure and each ACE, integrating features from brain measures or ACEs to create latent variables may be a better approach to detect small effects. We will discuss these points below.

There is a lack of consensus which experiences qualify as ACEs. Childhood adversity has been defined as “experiences that are likely to require significant adaptation by an average child and that represent a deviation from the expectable environment” (3). Also, ACEs have been defined as “childhood events, varying in severity and often chronic, occurring in a child’s family or social environment that cause harm or distress, thereby disrupting the child’s physical or psychological health and development” (61). An extensive body of research shows that experiences of maltreatment, sexual abuse and neglect impact the brain (26, 29, 62). However, in our study no data experiences of maltreatment, sexual abuse or neglect were available. Therefore, we focused on other types of ACEs. Our broad definition of ACEs results in only 17% of the children that were not exposed to any type of ACE and thus may not have fully captured the expected complexity and dimensionality of adversity. Because we did not assess the impact or severity of the ACE and did not include measures of functioning, it remains unclear how children experienced these events and whether the experiences are so disruptive that brain development or (future) functioning could be affected.

Another explanation for the small effects is that we study recent experiences, disregarding prenatal early life stress and ACEs that occurred more than a year ago. Effects of recent ACEs on brain structure may emerge later in development or even adulthood (15). It is possible that ACEs may impact brain structure over time, but effects on brain functioning may be easier to detect shortly after the experience. There are numerous studies that find a relation between ACEs and brain functioning during childhood or adolescence (26, 31, 62–64). For the effects that we found, it is plausible that they refer to an adverse family environment throughout childhood rather than a single event that occurred in the last year. For example, substance abuse problems in the family could have been present throughout childhood or even throughout pregnancy. No information on duration and severity of the ACEs was available and prenatal exposure to alcohol or drugs was not included in this study. In the same way, exposure to violence could be an indication of an adverse family environment in general or neighborhood disadvantage. To get a general idea of the broader social environment, we tested for difference in parental education, parental ethnicity, household income, child’s psychopathology and number of children in the household. We found that lower household incomes were more prevalent when parents reported exposure to violence and shorter parental education was related to substance abuse in the household.

A last point to consider is that there are different methodological approaches to deal with the small effect sizes when studying the relationship between childhood adversity and brain structure. In our study we opted for a broad approach to include regions beyond the traditionally studied fronto-limbic structures and to study each ACE separately. Our approach is similar to a recent study in 398 older adults that also included a large number of ROIs and different types of ACEs (10). In this study a similar pattern of subtle effects of ACEs on brain morphology was found. Another approach is to integrate brain measures (atlas-based or voxel-/vertex-wise) using multivariate techniques, e.g., non-negative matrix factorization (65), independent component analysis, canonical correlation analysis or partial least squares approaches (66). Principal component analysis was used in a study on the effects of brain structure and adverse lifetime experiences in adults (67). Another approach would be to improve the way that childhood adversity is measured, for example using extensive interviews to assess different ACEs and their impact (68). However, this approach is often not feasible in population-based cohorts. On the whole, statistical power remains a challenge when studying the effect of many ACEs on many brain morphology estimates in population cohorts.

For the main effects it remains unresolved if they are environmental, genetic or both. Neural effects in response to adversity could be adaptive in the short-term, but in the long-term these adaptations may contribute to risk or resilience. Lockdown restrictions during the COVID-19 pandemic can be seen as another example of an environmental stressor to some (69–73). All children in our study were measured before the onset of the pandemic, but pandemic-related ACEs may impact the follow-up data collected in the YOUth cohort study. Importantly, accelerated or delayed brain development could also be driven by genetic factors (74, 75) and the environment that parents can provide for their children is also influenced by genetics (76, 77).

Three limitations of this study were not yet discussed. One, MRI data were processed using adult templates while the use of age appropriate templates could have improved the registrations (78). Two, the group of children with missing MRI data scored slightly higher on the CBCL total problem scale and missing DWI data was more prevalent when children had a father with another self-reported ethnicity than Dutch. Three, the described cohort is homogenous with regard to self-reported ethnicity and on average participants have high socioeconomic status, and thus the sample is not representative of the general Dutch population (58) and results cannot readily be generalized to other parts of the world.

A better understanding of the impact of adversity on neural development is important given current pressing societal issues (79). Early detection of children that are at risk for negative outcomes later in life can help policy makers, health care professionals, families and schools to break with childhoods characterized by accumulated ACEs. Developmental neuroscience can play a crucial role to inform these interventions with regard to sensitive periods of development.

## Data availability statement

The datasets presented in this article are not readily available because the YOUth cohort study encourages and facilitates bona fide use of its data. Researchers who wish to use YOUth data, can find information about the data request procedure on our website. Processed data or scripts used in the current study can be requested through this procedure as well, but please feel to free to reach out to the corresponding author for support. Requests to access the datasets should be directed to https://www.uu.nl/en/research/youth-cohort-study/data-access.

## Ethics statement

The studies involving human participants were reviewed and approved by the Medical Research Ethics Committee Utrecht. Written informed consent to participate in this study was provided by the participants’ legal guardian/next of kin.

## Author contributions

EB, RB, and HH contributed to conception and design of the study. EB, PP, RB, and RM did the programming and data processing. EB, RB and HS contributed to the statistical analyses. HH provided resources. EB wrote the first draft of the manuscript including data visualization. All authors were involved in the design and quality assurance of the neuroimaging data, contributed to reviewing and revising the manuscript.

## Footnotes

1
https://fsl.fmrib.ox.ac.uk/fsl/fslwiki/TBSS/UserGuide

2
www.nitrc.org/projects/surfice/
