# Supplementary material for: Adverse childhood experiences and fronto-subcortical structures in the developing brain
Source: Front Psychiatry. 2022 Oct 6;13:955871. doi: 10.3389/fpsyt.2022.955871 (PMC9582338; doi:10.3389/fpsyt.2022.955871)
Supplement: Supplementary file 2 [file Data_Sheet_2.pdf]

Adverse childhood experiences and fronto-subcortical brain structures in YOUth

------- Supplement -------

Elizabeth E.L. Buimer[1*], Rachel M. Brouwer[1,2], René C.W. Mandl[1], Pascal Pas[1,3], Hugo G. Schnack[1,4], Hilleke E. Hulshoff Pol[1,5]

[1] UMC Utrecht Brain Center, University Medical Center Utrecht, Utrecht University, Utrecht, The Netherlands
[2] Department of Complex Trait Genetics, Centre for Neurogenomics and Cognitive Research, VU University Amsterdam, The Netherlands
[3] Experimental Psychology, Utrecht University, Utrecht, The Netherlands
[4] Department of Languages, Literature and Communication, Faculty of Humanities, Utrecht University, Utrecht, The Netherlands
[5] Department of Experimental Psychology, Helmholtz Institute. Utrecht University, Utrecht, The Netherlands

Corresponding author: Heidelberglaan 100, 3584CX Utrecht, The Netherlands. e.e.l.buimer@umcutrecht.nl

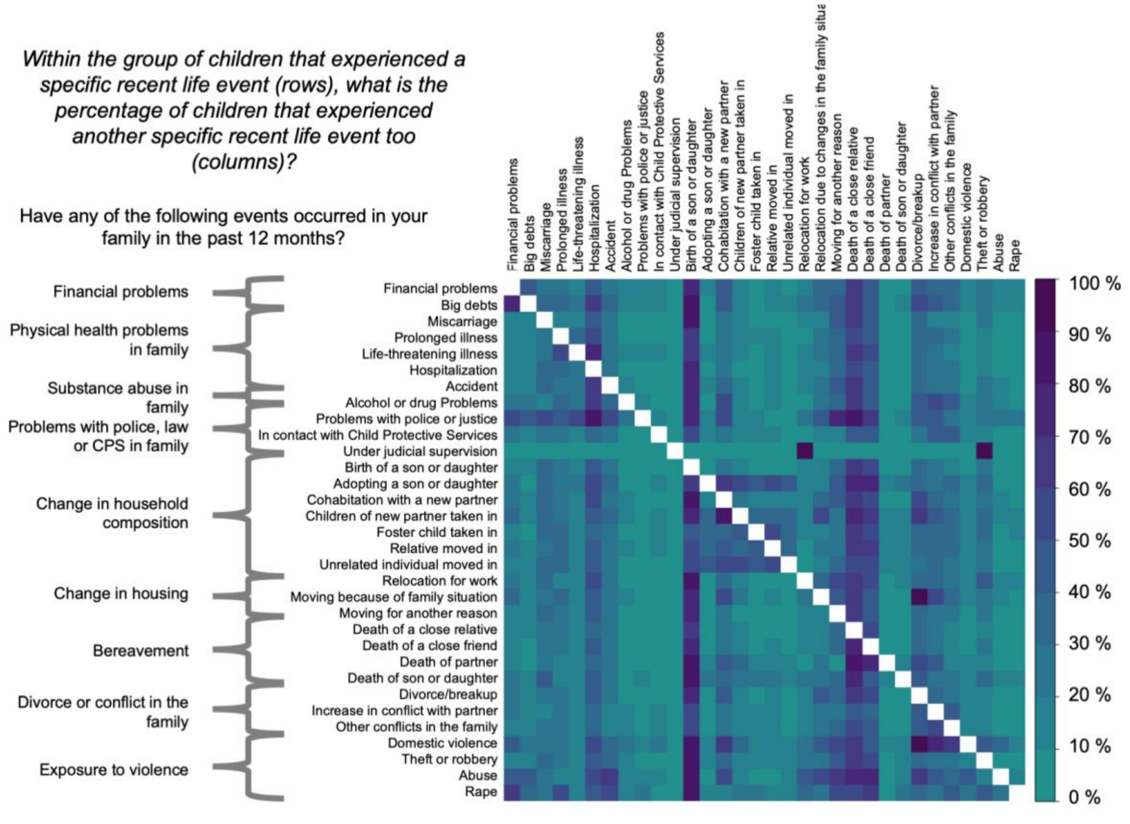

Figure S1. Overlap between scores on items of the recent life events questionnaire.

Figure S2. Bootstrap distribution for family exposure to violence and fractional anisotropy in the left cingulum bundle hippocampus region

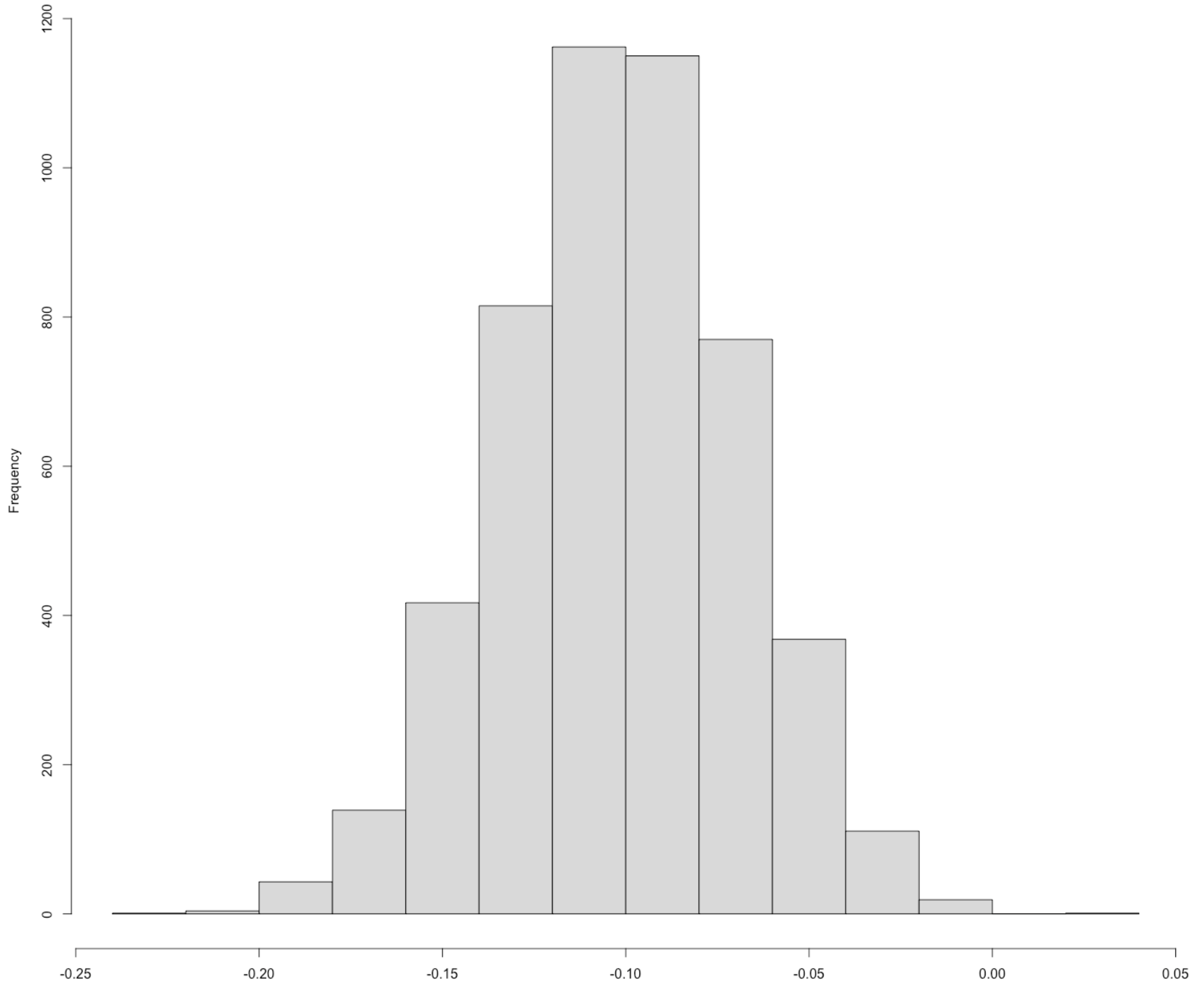

Table S6 Bootstrap results for family exposure to violence and fractional anisotropy in the left cingulum bundle hippocampus region compared to main results

	Beta [95% CI]	Bootstrapping mean (SD) [95% CI]
**ACE**	-.10 [-.17, -.04]	-.10 (0.03) [-.17, -.04]
**Age**	.05 [-.01, .12]	.05 (0.03) [-.01, .12]
**Sex**	-.09 [-.16, -.03]	-.09 (0.03) [-.16, -.03]
**DWI Acquisition**	-.49 [-.56, -.43]	-.49 (0.03) [-.55, -.44]

Figure S3. Bootstrap distribution for family exposure to violence and fractional anisotropy in the right cingulum bundle hippocampus region

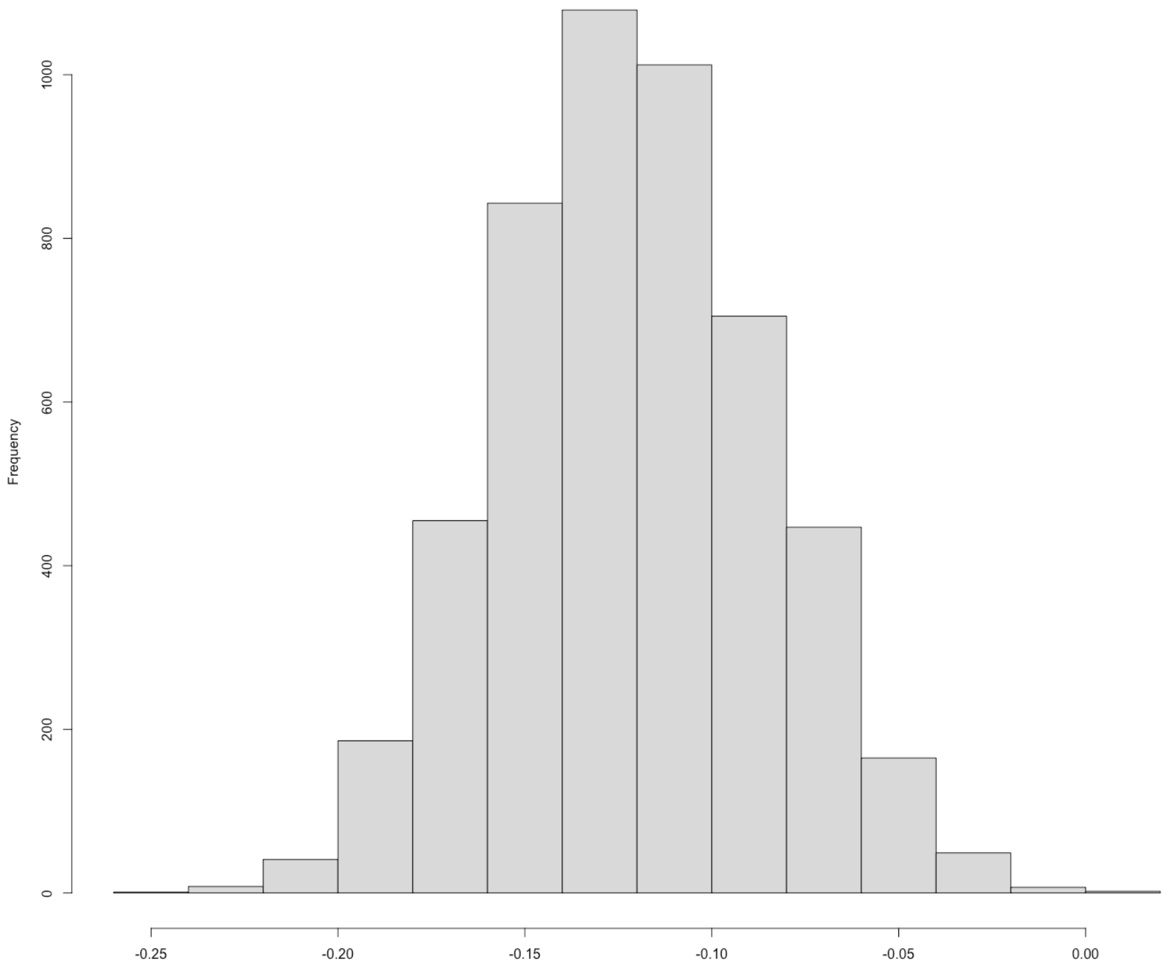

Table S7 Bootstrap results for family exposure to violence and fractional anisotropy in the right cingulum bundle hippocampus region compared to main results

	Beta [95% CI]	Bootstrapping mean (SD) [95% CI]
**ACE**	-.12 [-.19, -.05]	-.12 (0.03) [-.19, -.05]
**Age**	.07 [.00, .14]	.07 (0.04) [.00, .14]
**Sex**	-.04 [-.11, .03]	-.04 (0.04) [-.10, .04]
**DWI Acquisition**	-.30 [-.37, -.23]	-.30 (0.03) [-.37, -.24]

Figure S4. Bootstrap distribution for substance abuse in the household and cortical surface area in the left superior frontal gyrus

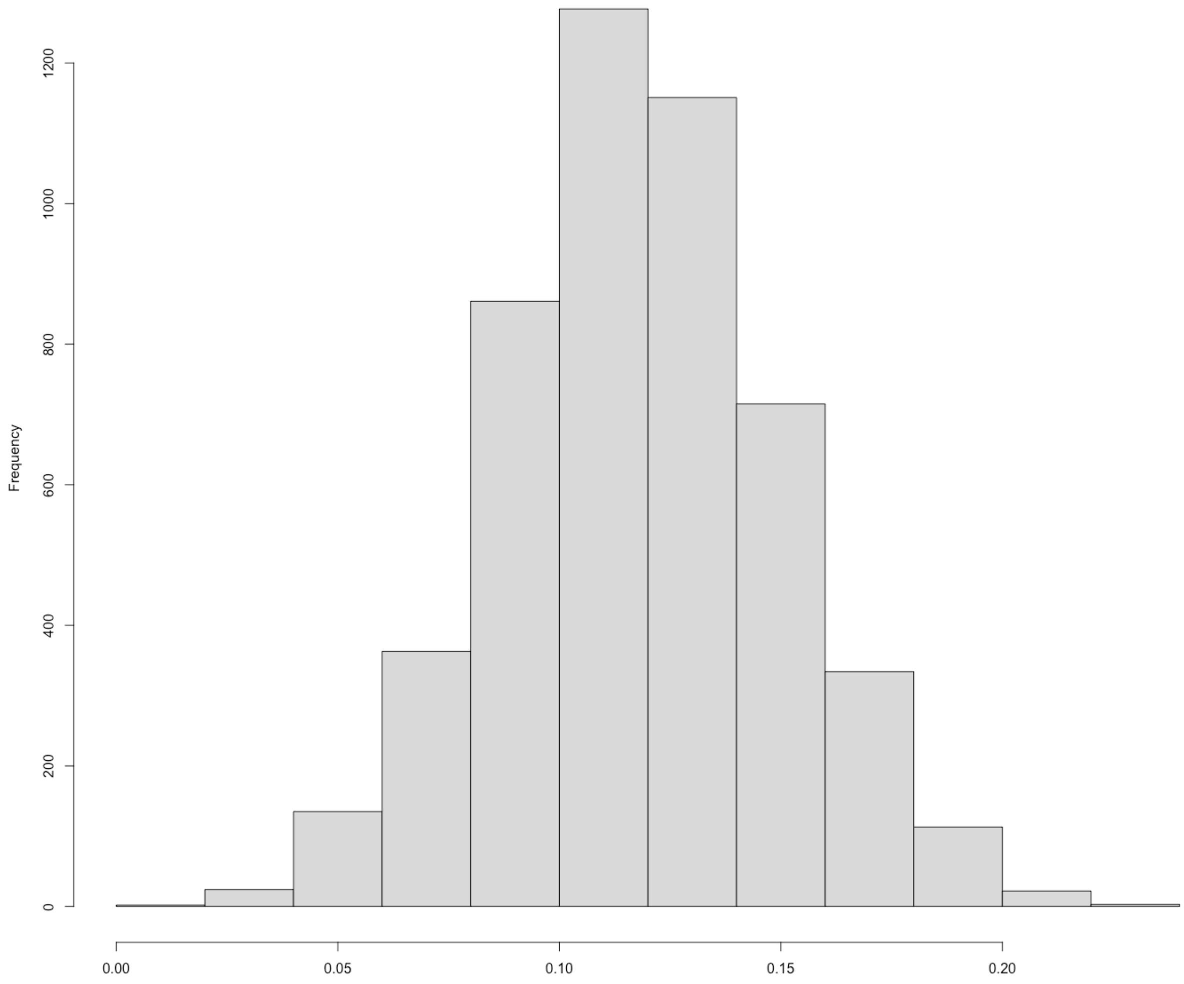

Table S8 Bootstrap results for substance abuse in the household and cortical surface area in the left superior frontal gyrus compared to main results

	Beta [95% CI]	Bootstrapping mean (SD) [95% CI]
**ACE**	.12 [.06, .18]	.12 (0.03) [.06, .18]
**Age**	.09 [.02, .15]	.09 (0.03) [.02, .15]
**Sex**	-.43 [-.50, -.37]	-.44 (0.03) [-.49, -.38]

Figure S5. Bootstrap distribution for substance abuse in the household and cortical surface area in the left pars triangularis

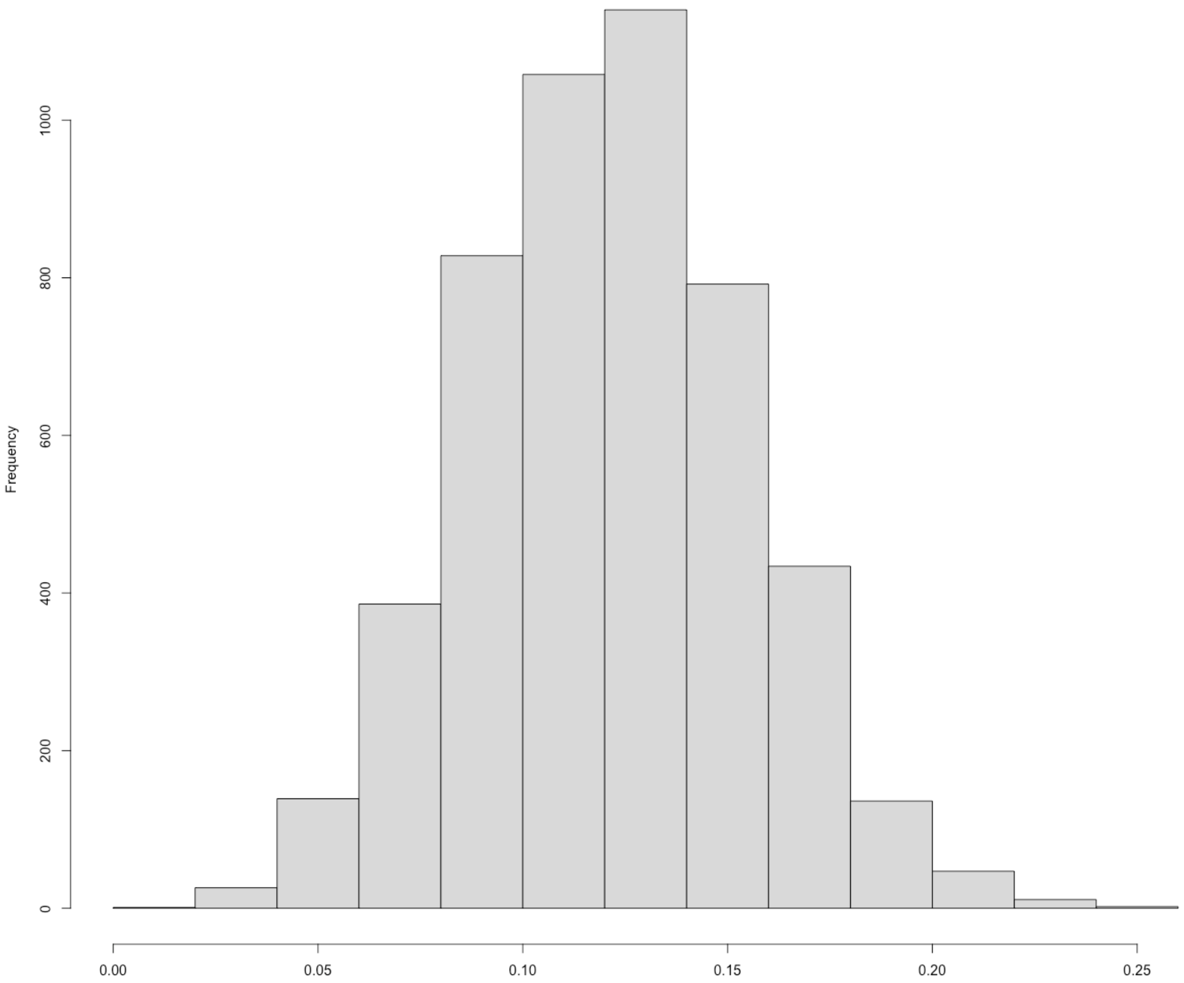

Table S9 Bootstrap results for substance abuse in the household and cortical surface area in the left pars triangularis compared to main results

	Beta [95% CI]	Bootstrapping mean (SD) [95% CI]
**ACE**	.12 [.06, .19]	.12 (0.03) [.06, .19]
**Age**	.07 [.01, .14]	.07 (0.03) [.01, .14]
**Sex**	-.32 [-.39, -.26]	-.32 (0.03) [-.38, -.26]

Figure S6. Bootstrap distribution for substance abuse in the household and cortical surface area in the right superior frontal gyrus

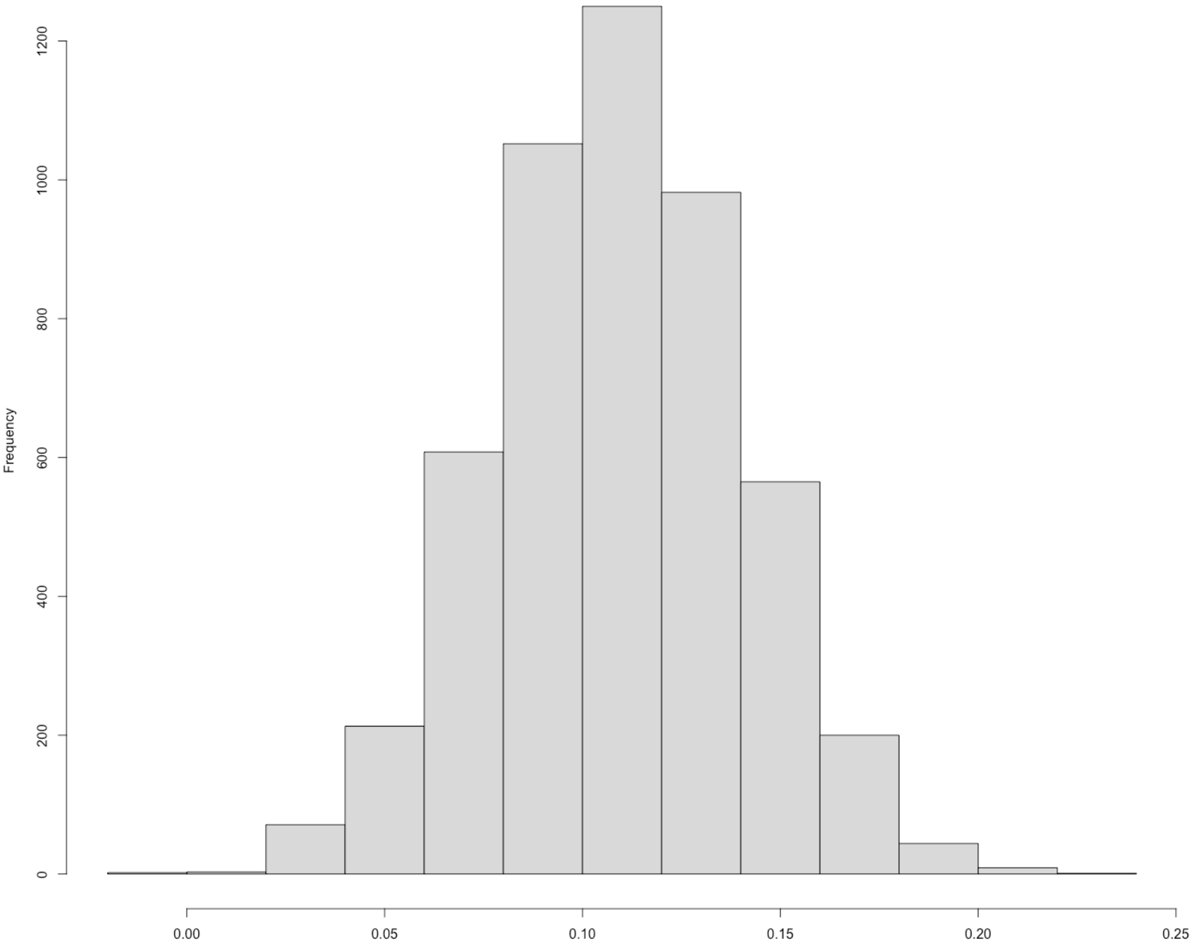

Table S10 Bootstrap results for substance abuse in the household and cortical surface area in the right superior frontal gyrus compared to main results

	Beta [95% CI]	Bootstrapping mean (SD) [95% CI]
**ACE**	.11 [.05, .17]	.11 (0.03) [.05, .17]
**Age**	.06 [-.00, .12]	.06 (0.03) [.00, .12]
**Sex**	-.43 [-.49, -.37]	-.43 (0.03) [-.48, -.37]

Figure S7. Bootstrap distribution for substance abuse in the household and cortical surface area in the left rostral middle frontal gyrus

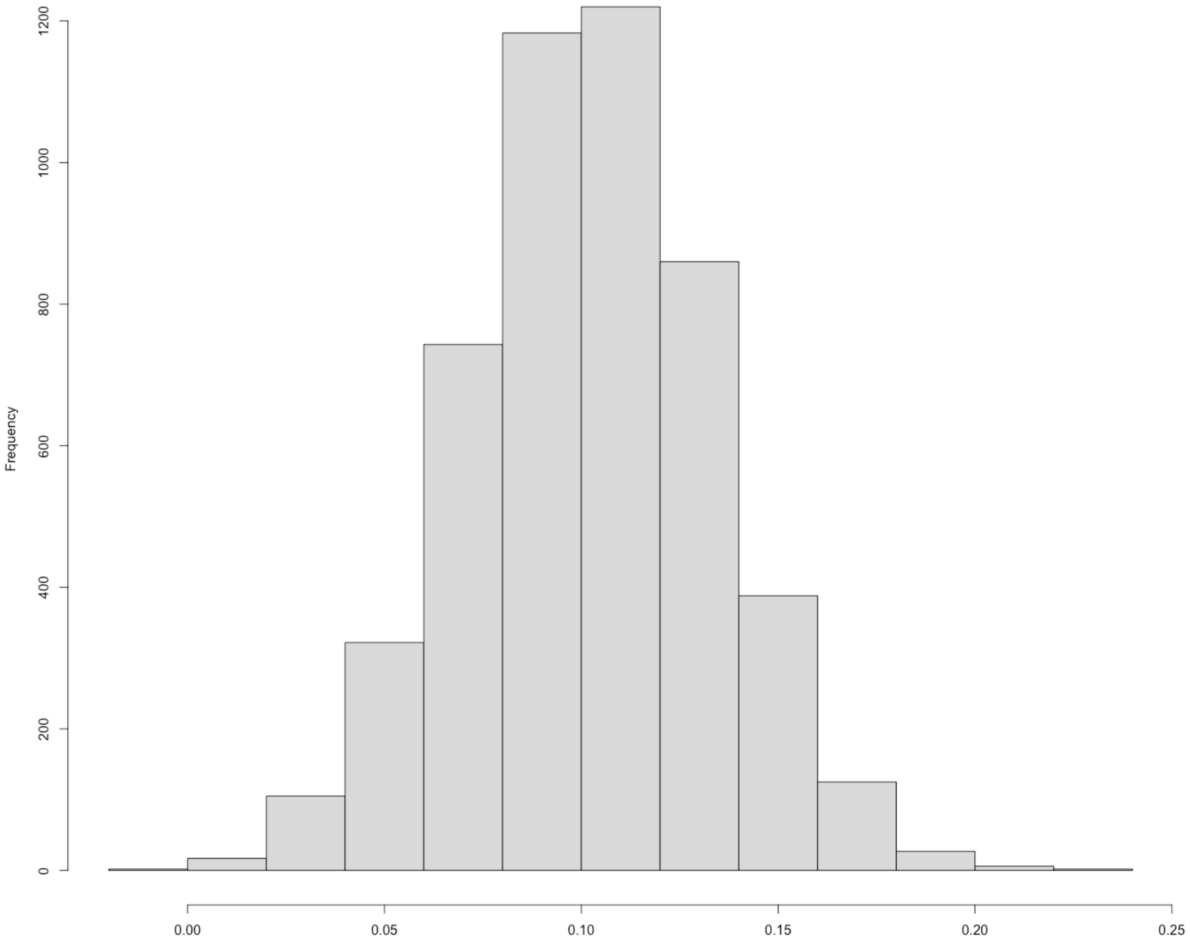

Table S11 Bootstrap results for substance abuse in the household and cortical surface area in the left rostral middle frontal gyrus compared to main results

	Beta [95% CI]	Bootstrapping mean (SD) [95% CI]
**ACE**	.10 [.04, .16]	.10 (0.03) [.04, .16]
**Age**	.09 [.03, .15]	.09 (0.03) [.03, .15]
**Sex**	-.43 [-.49, -.36]	-.43 (0.03) [-.48, -.37]

Figure S8. Bootstrap distribution for substance abuse in the household and cortical surface area in the right caudal anterior cingulate gyrus

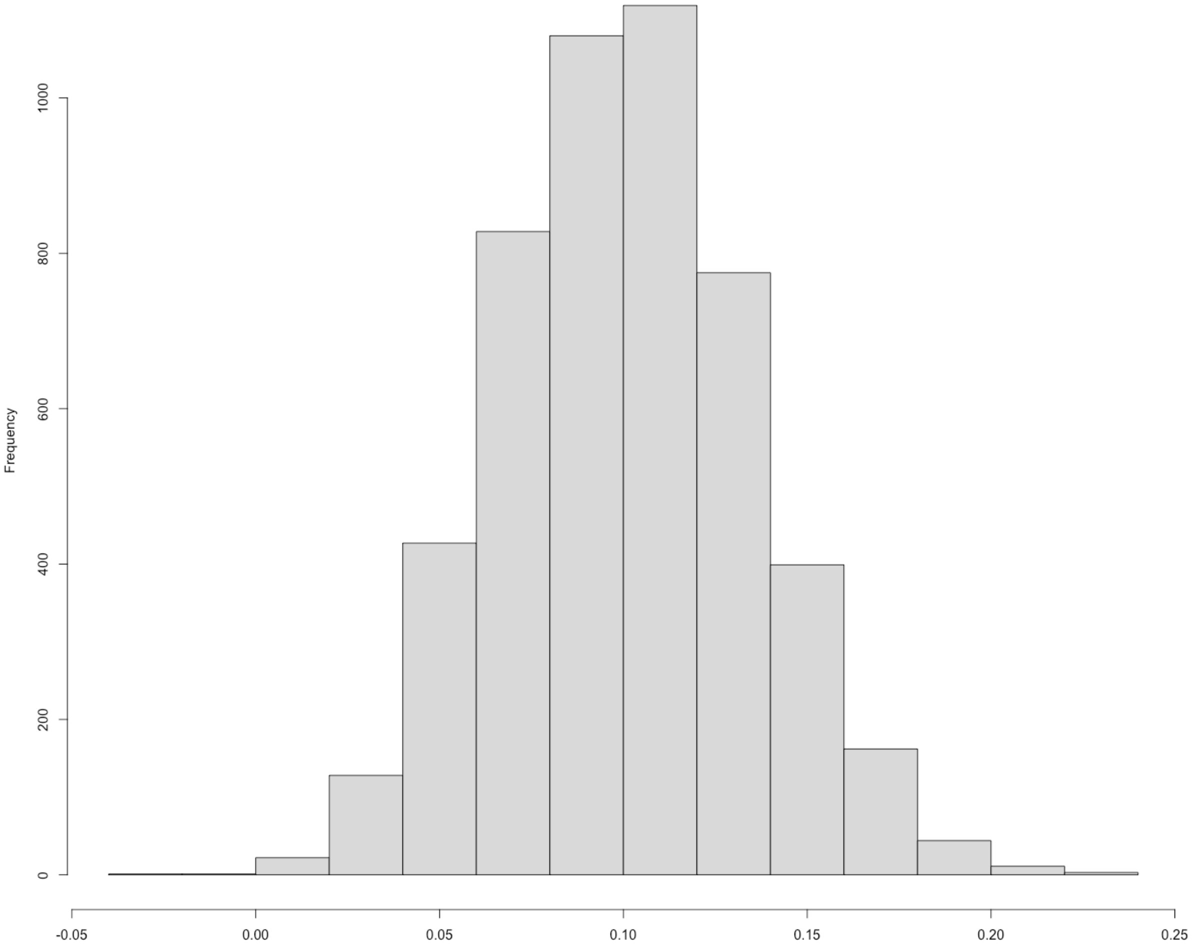

Table S12 Bootstrap results for substance abuse in the household and cortical surface area in the right caudal anterior cingulate gyrus compared to main results

	Beta [95% CI]	Bootstrapping mean (SD) [95% CI]
**ACE**	.10 [.03, .17]	.10 (0.03) [.04, .17]
**Age**	.07 [.00, .14]	.07 (0.03) [.01, .14]
**Sex**	-.25 [-.32, -.18]	-.25 (0.03) [-.32, -.18]

Supplementary method – Post-hoc analyses

Definitions

We used *mother* to indicate one parent or guardian (mostly female). We used *father* to indicate the second parent or guardian (mostly male). The *primary participating parent* is the parent or guardian that fills in the surveys when the report from only one parent is sufficient. The primary participating parents was the female parent in most cases.

CBCL total problem score

One parent filled out the CBCL to report on problem behavior of their participating child. The 118 specific problem items of the CBCL (0-to-2 scale) were summed for each child. Three additional open-ended questions allowed the respondent to fill out additional problems. We decided not to include these open-ended items in our sum score as most answers were overlapping with the specific problems and selecting valid problems from the answers was not feasible given our large sample size.

Parental self-reported ethnicity

Self-reported ethnicity was based on the question "*What is your ethnic background (multiple answers possible)?*". Multiple choice options were as followed:

1. *Dutch*
2. *European Union (excluding the Netherlands)*
3. *Turkish*
4. *European but not part of the European Union*
5. *Moroccan*
6. *African (excluding Morocco)*
7. *Surinamese*
8. *Antillean (from the former Netherlands Antilles, including Aruba, Bonaire, Curaçao, Saba, Sint Eustatius and Sint Maarten)*
9. *Latin American (excluding Suriname and the former Netherlands Antilles)*
10. *Indonesian*
11. *Asian (excluding Indonesia and Japan)*
12. *Other countries outside Europe (United States, Canada, Japan, Oceania)*

The multiple-choice options in our study were based on the former categorization by the Statistics Netherlands (CBS) based on countries that share specific migration history with the Netherlands and a distinction between 'western' and 'non-western' migration. From 2022 CBS uses a new classification system replacing the 'western' and 'non-western' main categories (https://www.cbs.nl/en-gb/longread/statistische-trends/2022/new-classification-of-population-by-origin). Given the small prevalence of parents with a migration background in our cohort and to not be bound by the categorization system above, we decided to indicate if parents had at least one other self-reported ethnicity in addition to or instead of Dutch.

Parental education level

Educational attainment was calculated by conversing the highest completed education to education in years to facilitate international comparison:

- *Primary education (BAO) – 6 years*
- *Special primary education (SBAO) – 6 years*
- *Education abroad comparable to primary education in the Dutch school system – 6 years*

- *(Secondary) Special education ((V)SO) – 10 years*
- *Practical education (PRO) – 10 years*
- *Secondary vocational education ‘basis/kader’ (VMBO-BK) – 10 years*
- *Secondary vocational education ‘theoretische leergang’ (VMBO-TL) – 10 years*
- *Higher general secondary education (HAVO) – 11 years*
- *Higher secondary education (VWO) – 12 years*
- *Secondary vocational education (MBO) – 14 years*
- *Education abroad comparable to practical or secondary vocational education in the Dutch school system – 12 years*
- *Applied university (HBO) – 15 years*
- *Education abroad comparable to applied university in the Dutch school system – 15 years*
- *University education (WO) – 17 years*
- *Education abroad comparable to higher vocational education or university in the Dutch school system – 17 years*

Monthly household income

Similar to the study by Fakkel et al. (2020), monthly household income was based on the answer of the primary participating parent on the question: *“Can you make an estimation of [if single] your gross income or [if not single] the total gross household income?”.* Options “*prefer not to say*” and “*can’t estimate*” were not included in the tables and percentages.

Number of children at home

Both parents were asked about the composition of their household. We assumed that the answers of the primary participating parent were most indicative of the number of children that the participating child grows up around. Therefore, we created a sum score based on the answers of the participating parent.

Who do you live with? (multiple answers possible):

- *Indicate the number of biological children*
- *Indicate the number of foster children*
- *Indicate the number of adopted children*
- *Indicate the number of stepchildren*

Next, the sum scores were classified to 0 or 1; 2; over 3 children at the home of the participating parent.

Statistics

To compare the main sample versus the subsets (Table S13 and S14) and the children with versus without ACEs (Table S15 and Table S16), we used Chi-squared tests for the categorical variables and the Mann-Whitney U test (non-parametric because of skewness) for the CBCL and the education in years. We used an alpha level of .05 to test for significance.

Reference

Fakkel, M., Peeters, M., Lugtig, P., Zondervan-Zwijnenburg, M. A. J., Blok, E., White, T., ... & Vollebergh, W. A. M. (2020). Testing sampling bias in estimates of adolescent social competence and behavioral control. Developmental Cognitive Neuroscience, 46, 100872.

Table S13 Responders versus non-responders for T1 and life events data

	**T1 and life events survey available**	
	**Yes**	**No - missing**
Sex (% girls)	*N=785*	*N=170*
	56	56
Mean age in years (SD)	*N=785*	*N=170*
	9.56 (0.85)	9.44 (0.89)
Mean CBCL total problems (SD)*	*N=642*	*N=413*
	22.17 (16.42)	24.10 (16.54)
Self-reported ethnicity mother (%)	*N=740*	*N=472*
Dutch	92	90
Dutch and another ethnicity	2	3
Another ethnicity	6	7
Self-reported ethnicity father (%)	*N=592*	*N=363*
Dutch	94	91
Dutch and another ethnicity	2	2
Another ethnicity	3	6
Mean education in years mother (SD)	*N=740*	*N=472*
	15.21 (1.92)	14.97 (2.11)
Mean education in years father (SD)	*N=592*	*N=363*
	14.91 (2.47)	14.76 (2.54)
Gross monthly household income (%)	*N=690*	*N=443*
< €1.200	2	1
€1.200 - €2.000	5	7
€2.000 - €3.200	9	7
€3.200 - €4.000	17	20
> €4.200	68	64
Number of children at home (%)	*N=750*	*N=472*
0 or 1	11	13
2	54	51
3 or more	35	35

* significant Mann-Whitney U test.

Table S14 Responders versus non-responders for DWI and life events data

	DWI and life events survey available	
	**Yes**	**No - missing**
Sex (% girls)	*N=702*	*N=253*
	57	55
Mean age in years (SD)	*N=702*	*N=253*
	9.55 (0.85)	9.49 (0.88)
Mean CBCL total problems (SD)*	*N=578*	*N=477*
	22.17 (16.70)	23.84 (16.20)
Self-reported ethnicity mother (%)	*N=660*	*N=552*
Dutch	91	90
Dutch and another ethnicity	2	3
Another ethnicity	7	7
Self-reported ethnicity father (%)**	*N=531*	*N=424*
Dutch	95	91
Dutch and another ethnicity	2	2
Another ethnicity	3	6
Mean education in years mother (SD)	*N=660*	*N=552*
	15.21 (1.93)	15.00 (2.08)
Mean education in years father (SD)	*N=531*	*N=424*
	14.98 (2.41)	14.71 (2.60)
Gross monthly household income (%)	*N=617*	*N=548*
< €1.200	1	2
€1.200 - €2.000	5	7
€2.000 - €3.200	8	8
€3.200 - €4.000	17	20
> €4.200	69	64
Number of children at home (%)	*N=669*	*N=563*
0 or 1	11	13
2	54	52
3 or more	35	34

* significant Mann-Whitney U test.

** significant χ^2 test.

Table S15 Demographics for children with and without experiences of substance abuse in the household

	Substance abuse in the household	
	**Yes**	**No**
Sex (% girls)	*N=15*	*N=770*
	53	56
Mean age in years (SD)	*N=15*	*N=770*
	9.52 (0.84)	9.56 (0.85)
Mean CBCL total problems (SD)	*N=10*	*N=632*
	17.20 (7.19)	22.24 (16.51)
Self-reported ethnicity mother (%)	*N=14*	*N=726*
Dutch	86	92
Dutch and another ethnicity	7	2
Another ethnicity	7	6
Self-reported ethnicity father (%)	*N=13*	*N=579*
Dutch	100	94
Dutch and another ethnicity	0	2
Another ethnicity	0	3
Mean education in years mother (SD)*	*N=14*	*N=726*
	14.07 (2.23)	15.23 (1.91)
Mean education in years father (SD)*	*N=13*	*N=579*
	13.38 (2.96)	14.95 (2.45)
Gross monthly household income (%)	*N=1*	*N=679*
< €1.200	9	1
€1.200 - €2.000	0	5
€2.000 - €3.200	9	9
€3.200 - €4.000	27	17
> €4.200	55	68
Number of children at home (%)	*N=14*	*N=736*
0 or 1	14	11
2	64	54
3 or more	21	35

* significant Mann-Whitney U test.

Table S16 Demographics for children with and without family exposure to violence

	Exposure to violence	
	**Yes**	**No**
Sex (% girls)	*N=80*	*N=622*
	66	56
Mean age in years (SD)	*N=80*	*N=622*
	9.54 (0.86)	9.55 (0.85)
Mean CBCL total problems (SD)	*N=68*	*N=510*
	24.06 (20.98)	21.91 (16.05)
Self-reported ethnicity mother (%)	*N=76*	*N=584*
Dutch	86	92
Dutch and another ethnicity	4	2
Another ethnicity	11	6
Self-reported ethnicity father (%)	*N=67*	*N=464*
Dutch	93	95
Dutch and another ethnicity	4	2
Another ethnicity	3	3
Mean education in years mother (SD)	*N=76*	*N=584*
	15.28 (2.03)	15.20 (1.92)
Mean education in years father (SD)	*N=67*	*N=464*
	14.75 (2.78)	15.01 (2.36)
Gross monthly household income (%) *	*N=69*	*N=548*
< €1.200	6	1
€1.200 - €2.000	6	5
€2.000 - €3.200	4	9
€3.200 - €4.000	16	17
> €4.200	68	69
Number of children at home (%)	*N=76*	*N=593*
0 or 1	18	10
2	58	53
3 or more	24	37

* significant χ^2 test.
